# Genome Editing and Muscle Stem Cells as a Therapeutic Tool for Muscular Dystrophies

**DOI:** 10.1007/s40778-017-0076-6

**Published:** 2017-04-24

**Authors:** Veronica Pini, Jennifer E. Morgan, Francesco Muntoni, Helen C. O’Neill

**Affiliations:** 1 Molecular and Developmental Neurosciences Program, The Dubowitz Neuromuscular Centre, UCL Great Ormond Street Institute of Child Health, 30 Guilford Street, London, WC1N 1EH UK; 2 Embryology, IVF and Reproductive Genetics Group, Institute for Women’s Health, University College London, 86-96 Chenies Mews, London, WC1E 6HX UK

**Keywords:** Stem cells, Muscular dystrophies, CRISPR/Cas, Genome editing, Gene therapy, Precision medicine

## Abstract

**Purpose of Review:**

Muscular dystrophies are a group of severe degenerative disorders characterized by muscle fiber degeneration and death. Therapies designed to restore muscle homeostasis and to replace dying fibers are being experimented, but none of those in clinical trials are suitable to permanently address individual gene mutation. The purpose of this review is to discuss genome editing tools such as CRISPR/Cas (clustered regularly interspaced short palindromic repeats/CRISPR-associated), which enable direct sequence alteration and could potentially be adopted to correct the genetic defect leading to muscle impairment.

**Recent Findings:**

Recent findings show that advances in gene therapy, when combined with traditional viral vector-based approaches, are bringing the field of regenerative medicine closer to precision-based medicine.

**Summary:**

The use of such programmable nucleases is proving beneficial for the creation of more accurate in vitro and in vivo disease models. Several gene and cell-therapy studies have been performed on satellite cells, the primary skeletal muscle stem cells involved in muscle regeneration. However, these have mainly been based on artificial replacement or augmentation of the missing protein. Satellite cells are a particularly appealing target to address these innovative technologies for the treatment of muscular dystrophies.

## Introduction

Among the many tissues that take part in the formation of the human body, skeletal muscle contributes to almost 50% of our body mass. Skeletal muscle formation, or myogenesis, is an active process that begins in embryonic development. Muscle growth and regeneration can occur throughout the entire lifespan to ensure its fundamental role in structural support and posture maintenance, breathing, thermoregulation, and metabolism.

**Fig. 1:**
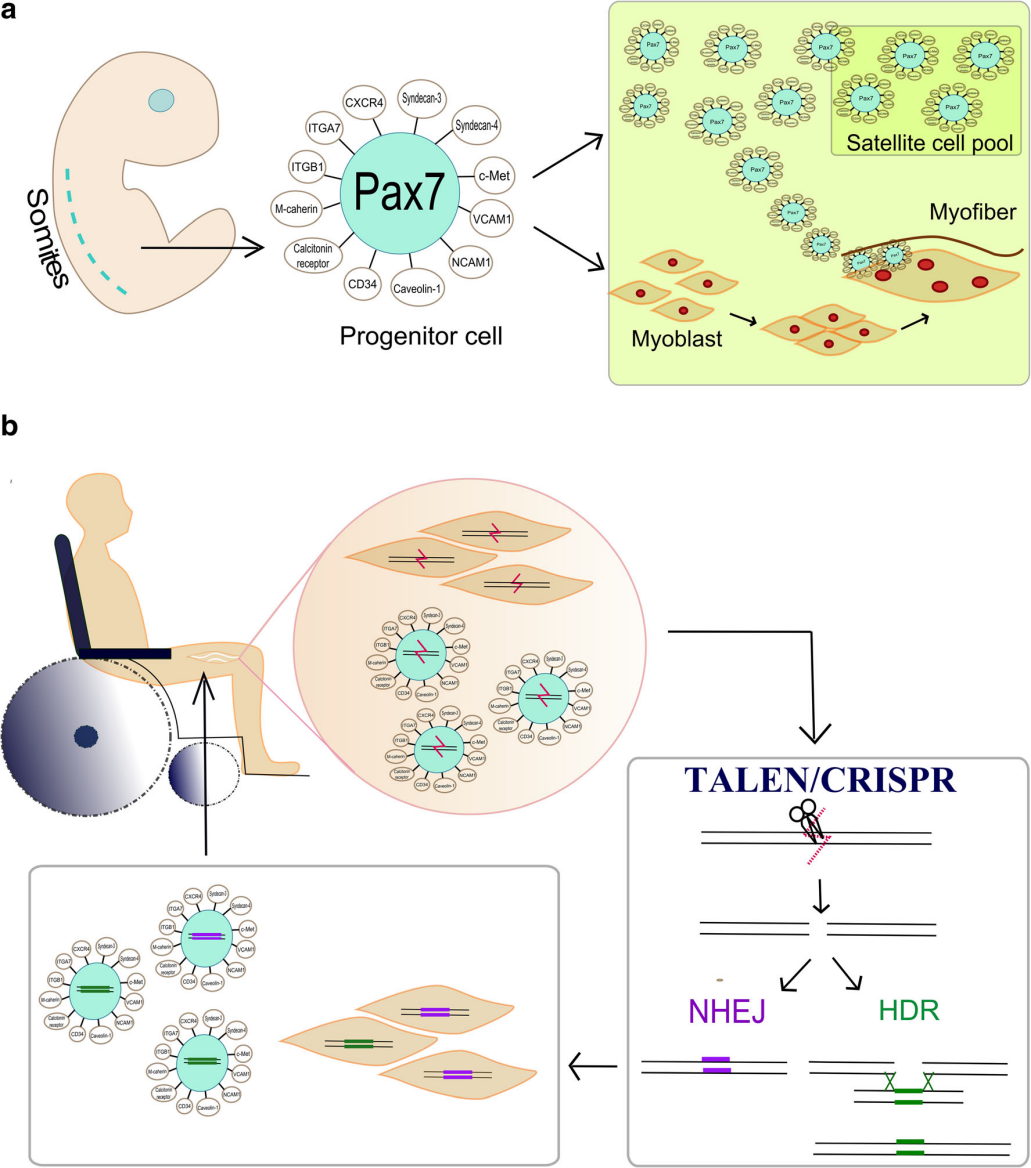
**a** Satellite cells originate from dorsal somites and are characterized by the expression of intracellular and extracellular markers: Pax7, CXCR4, Syndecan 3- and -4, c-Met, VCAM1, NCAM1, Caveolin-1, CD34, Calcitonin receptor, M-Cadherin. Integrin-α7 (ITGA7) and integrin-β1 (ITGB1). Satellite cells generate both cells aimed to replenish the satellite cell pool and cells that develop into myoblasts, precursors of the muscle fiber. Satellite cells reside at the periphery of the muscle fiber. **b** Diagram of regenerative medicine: satellite cells isolated from patient muscle and satellite-cells derived myoblasts can be treated with engineered nucleases (TALEN/CRISPR) to introduce a double strand break in their genome sequence, thus eliminating the mutation. NHEJ or HDR re-join the cleaved DS break, restoring the sequence. Corrected cells are then transplanted back into patient’s muscle

The functional unit of skeletal muscle is the myofibre. This is an elongated and multinucleated cell derived from the fusion of undifferentiated muscle precursors, known as myoblasts. During myogenesis, skeletal muscle stem cells (satellite cells) are generated (Fig. 1 a). Satellite cells are located underneath the basal lamina of the muscle fiber and contribute to muscle growth, maintenance, and regeneration. Satellite cells are normally quiescent in adult muscle until a stimulus or damage that can arise from either physiological conditions (exercise [1], aging [2]) or diseases (cachexia [3], sarcopenia [4], muscular dystrophies [5]) activates them to trigger muscle regeneration.

Upon activation, satellite cells generate myogenic cells called myoblasts, that either fuse together to form myotubes that mature into new myofibers or repair damaged segments of existing myofibres [6].

Muscular dystrophies are genetically inherited pathologies characterized by extensive muscle fiber damage that leads to the activation of satellite cells and to the exhaustion of their pool, with consequent impairment of the regenerative process. There are, at present, no therapies available to address muscle degeneration.

Regenerative medicine aims to combine gene- and cell-therapy approaches to restore the genetic defects in diseased cells, tissues, and organs in order to re-establish their functionality. In muscle, regeneration can be implemented by isolating patient-derived cells, correcting and transplanting them back into the damaged tissue. However, none of the gene therapy approaches designed to date can be expanded to correct the heterogeneous variety of mutations responsible for each muscular dystrophy subtype, limiting their applicability to a subset of patients only.

### Satellite Cells: Origin and Characterization

The ability of muscle fibers to repair damage and regenerate comes from the presence of stem cell populations that cooperate in the myogenic process. Among them, satellite cells have proven to be essential and indispensable in the regeneration of muscle [7–10]. This stem cell population was first discovered in 1961 by Alexander Mauro and Robert Katz [11, 12] who, in two independent studies, identified cells located between the plasma membrane and the basement membrane of skeletal muscle fibers isolated from frogs and rats. Harunori Ishikawa later observed that this defined peripheral location is conserved in most mammalian species and named them satellite cells [13]. Muscle satellite cells have distinct origins that correlate to the particular muscle they will generate; apart from head muscles, most of the skeletal muscles originate from the dorsal aspect of the somites in a process tightly controlled by the expression of muscle-specific transcription factors that vary dynamically according to specific cell-cycle phases [14]. Embryonically, dermomyotome cells, committed to myogenic development, express the intracellular markers Paired-box domain transcription factors Pax3 and Pax7, which are crucial regulators of trunk and limb muscle formation [15] as they act upstream of hierarchically related muscle regulatory factors (MRFs). Specifically, Pax3 expression appears to be indispensable during early embryonic development, but is less expressed in adult muscle [16]. Pax7 instead, takes the lead in later gestational stages and in postnatal muscle growth [17], and as it is expressed both in quiescent and activated satellite cells, it is considered to be a universal marker [18]. Cells expressing high levels of Pax7 are characterized by lower metabolic rate and lower division rate, while a low level of Pax7 correlates with higher myogenin levels [19].

Other than their expression of Pax7 and location at the periphery of the fiber, satellite cells are defined by a high ratio of nucleus-to-cytoplasm and cell surface markers: M-cadherin, α7- and β1- integrins, tyrosine receptor kinase c-Met (regulated by Pax3), CXCR4 (C-X-C chemokine receptor type 4), syndecan-3 and-4, calcitonin receptor, caveolin-1, CD34, VCAM1 (vascular cell adhesion molecule-1) and NCAM1 (neural cell adhesion molecule-1) [20, 21] Barx2 as well as nuclear envelope proteins laminA/C and Emerin [21, 22]. Since these markers can also be present in other cells, selection of a combination of specific markers can be used to isolate pure satellite cell pools via fluorescence-activated cell sorting (FACS): these are defined through positive selection of cells expressing both α7-integrin and CD34, combined with negative selection of CD45 (marker of hemotopoietic lineage), CD11b (leukocytes), CD31 (leukocytes and endothelial cells), and Sca-1 (mesenchymal and hematopoietic stem cells) [23]. Alternatively, satellite cells can be isolated by physically stripping them from myofibres [24, 25].

A broad range of heterogeneity exists within the satellite stem cell population. There is evidence that there are two subpopulations of satellite cells that are functionally distinct; one is responsible for muscle growth, is present in higher amount in males and decreases with aging, while the other, whose task is muscle regeneration after injury, is not sex-related and is maintained with age [26]. Satellite cells differ in fast and slow muscles, as those residing in fast muscles tend to form fast myosin isoforms, compared to those residing in slow muscles that form slow myosin isoforms [27].

Also, satellite cells undergo asymmetric divisions from which arise both cells committed to myogenic differentiation (myoblasts) and cells destined to replenish the quiescent satellite cell pool [28]: a subpopulation of cells, characterized by differential markers and Myf5 regulatory factor expression, showed a reduced division rate and higher engraftment capacity in a mouse model and is thought to be responsible for the maintenance of the satellite cell pool, as opposed to the remaining population which is more prone to differentiation [10].

In vitro and in vivo studies have also highlighted differences in satellite cells derived from muscles having different embryonic origin. For example, Pax3 is highly expressed in the diaphragm and trunk muscles together with Pax7, but is not expressed in the hindlimbs [29]. Diaphragm satellite cells exhibit greater replicative potential compared to limb, trunk, and facial muscle, though are limited in their ability to differentiate [30]. A chronic proliferation is also observed in laryngeal, pharyngeal, and extraocular satellite cells, possibly due to a subpopulation of highly active satellite cells [31–33]. Delayed differentiation seems to correlate with impaired regenerative ability in masseter muscles following acute injuries, despite the increase in number of satellite cells present in this muscle with age [34]. However, the method with which different satellite cell profiles contribute to the specific involvement of preferential muscles in distinct pathologies is still matter of debate in the scientific community.

### Muscle Regeneration

Normally, satellite cells in adult muscles lie in their niche in a quiescent state until a stimulus “awakens” the dormant cells and stimulates them to proliferate and differentiate into mature myofibers.

Upon stress or damage, the niche and fibro/adipogenic precursors [35] in the fiber provide stimuli to promote muscle regeneration. The muscle fiber releases molecules, responsible for the inflammatory response, that recruit neutrophils and macrophages that remove the cellular debris, while leaving the basal lamina intact as a scaffold for muscle regeneration [36, 37]. Fibro-adipogenic precursors and connective tissue-fibroblasts [9] are also involved in the regenerative process, as they release pro-differentiation signals that help muscle healing [35]. Meanwhile, the fiber rapidly produces growth factors, chemokines, and cytokines and releases them both systematically and locally to activate satellite cells and stimulate them to proliferate and undergo asymmetric divisions [38–40].

The regenerative process largely recapitulates muscle fiber development, as both are characterized by differential expression of four essential myogenic regulatory factors (MRFs), hierarchically expressed: Myf5, MyoD, MRF4, and myogenin [41]. In early myogenesis, Myf5 is the first to be expressed followed by MyoD. Their combined expression plays a critical role in muscle formation, as shown in double Myf5-and-MyoD knock-out mice where skeletal muscle is not detected [42]. These two factors act upstream of MRF4 and myogenin which are essential for late [43] and terminal myofiber differentiation, respectively [44].

Similarly, quiescent satellite cells characterized by the expression of Pax7/Myf5 proliferate to become myoblasts that also express MyoD. During the process of myogenic differentiation into mature myofibers, instead, the expression of Pax7 and Myf5 decreases, levels of myogenin and MRF4 increase. Finally, MyoD levels are reduced when mature myoblasts fuse together to give rise to new myofibers, in which contractile proteins (like myosin heavy chain, among others) start to be expressed.

Other than MRFs, the differential expression of many other genes controls the activation status of satellite cells: indeed, the maintenance of quiescence involves more than 500 genes, mainly responsible for the control of cell-cycle, extra-cellular matrix formation, and interaction among cells [20]. Cell-cycle inhibition is determined by Foxo3 and Notch: The Foxo3 transcription factor prevents cell cycle entry and suppresses terminal differentiation by activating Notch [45], that in turn, acts as an inhibitor of the p38/MAPK and JNK pathways necessary for self-renewal [46, 47]. Moreover, the cell-cycle is arrested by the overexpression of the cyclin-dependent kinases inhibitor p27kip1 and Sprouty1, an inhibitor of MAPK/ERK pathway [48]. The satellite cell microenvironment holds satellite cells in quiescence through the interaction of proteins like M-cadherin [49] and α7- and β1-integrins expressed both on the cell surface and in the niche [50]. The integrity of the niche itself is also ensured by the expression of metalloproteinase inhibitors to avoid extracellular matrix degradation [51].

Conversely, upon activation, satellite cells first activate the p38/MAPK pathway leading to Myf5 and MyoD transcription factor expression and to subsequent entry in the cell cycle. The downregulation of Sprouty1 and the increased expression of TNF alpha, cytokines, and other growth factors released by the damaged fibers [39, 52, 53] also activate Ekr1/2 and JNK pathways, while inhibiting the JAK-STAT pathway. The Wnt4/β-catenin pathway is temporarily activated, but after a few replicative cycles is downregulated to limit the regenerative process and, together with p21^Kip1^, p19^Arf^, and p57 cyclin-dependent kinases, favors differentiation [54–56].

### Muscular Dystrophies

Muscular dystrophies are a heterogeneous group of rare inherited neuromuscular disorders characterized by progressive muscle weakness and muscle degeneration. They are clinically, genetically, and biochemically heterogeneous, having different ages of onset and causing either partial impairment in mobility or more severe consequences that lead to an early death [57•]. They derive from mutations in genes coding for either cytoplasmic, nuclear, membrane, or extracellular matrix proteins essential for muscle function and homeostasis [58]. Muscular dystrophies are grouped in nine main categories with multiple subgroups, each defined by a particular pattern of muscle weakness distribution and clinical severity dependent on the location and type of mutation [57•].

Regardless of the affected protein, multifactorial events lead to a common outcome for all muscular dystrophies: the perturbation of the fiber microenviroment by chronic damage which causes an inflammatory response that exacerbates fiber degeneration and its replacement by non-functional adipose and fibrotic scar tissue.

In some muscular dystrophies, the level of the impairment clinically observed seems to relate to the inability of satellite cells to effectively contribute to muscle regeneration [59]. Satellite cells become activated to combat the fiber degeneration; however, each cell cycle shortens the telomeres of the satellite cells and contributes to cell senescence [60, 61], so that the pool is rapidly exhausted.

Another obstacle to effective muscle regeneration is presented by the environment in which the regenerative process takes place: even if a satellite cell itself retains its regenerative capacity, the dystrophic niche may be hostile and unfavorable for permitting efficient regeneration [62, 63, 64••].

Moreover, if satellite cells express the mutated gene, regeneration may be further impaired from the early stages [65]. One example of this scenario is provided by the severe Duchenne Muscular Dystrophy (DMD), caused by mutations in the dystrophin gene located on the X chromosome, where it spans 2.2 megabases of DNA [66].

Dystrophin is a membrane-associated protein whose task is to provide a link between the sarcolemma and cytoplasmic actin; if absent, myofibers undergo necrosis following repeated contractions.

Three main kinds of mutations in dystrophin cause DMD: deletions, duplications, and point mutations [67]. Large intragenic deletions and duplications account for about 65 and 10% of cases, respectively, while nonsense mutations and other small mutations (splice site, small insertions, small deletions, inversions) cover the remaining 25%. Although rearrangements can occur anywhere in the dystrophin gene, the deletions and duplications are located mainly in two hotspots, the main one being at the 3′ of the gene (exons 44–53) and the other at the 5′ (exons 3–19).

In satellite cells, there is evidence that dystrophin controls the determination of their polarity and asymmetric divisions: the number of muscle progenitors might therefore be decreased in DMD, limiting the regenerative lifespan [68] and contributing to its early exhaustion. Finally, the defective myoblast differentiation and fusion reported by Delaporte and Jasmin in 1984 [69, 70] could be considered to impede the regenerative process.

To date, there are no therapies available to treat muscular dystrophies. Corticorsteroids are provided as a palliative therapy that can hinder muscle degeneration by interfering with immune system activation and inflammation arising from muscle breakdown [71–73]. However, this approach does not completely stop the disease progression, nor heal the genetic defect, and is associated with considerable long term side effects.

Genome engineering is a promising alternative strategy, since by exploiting different nucleases and subsequent pathways of DNA repair, it would allow the design of therapies tailored for each specific mutation causing DMD.

## Designing the Path to Precision Medicine: Satellite Cells Meet CRISPR

### The Rise of Genome Engineering Tools

“Genome editing” or “genome engineering” has recently emerged as a promising gene therapy approach to allow the introduction of efficient and precise genetic alterations in a variety of cells [74]. This technology relies on the use of programmable nucleases. These are hybrid proteins composed of domains able to bind DNA in a sequence-specific fashion, fuse to nonspecific DNA cleavage modules, and can therefore be addressed to introduce double-strand breaks (DSB) at any targeted genomic locus. DSBs and the further cell repair mechanisms that are stimulated to restore the integrity of the genetic information [75] can be exploited to introduce precise mutations.

In eukaryotic cells, DSBs are repaired via two distinct pathways: homology-directed repair (HDR) and non-homologous end joining (NHEJ) [76]. HDR pathway occurs only at defined cell cycle stages (during late S phase or G2) and requires the presence of an identical, or almost identical, sequence to be used as repair template, while NHEJ involves the simple ligation of the two DNA ends [77] in an error-prone way that generates unfaithful repair products derived by the introduction of small deletions and/or insertions. The two pathways are responsible for different genomic alterations when applied to gene editing: HDR is exploited to insert a specific mutation in the genome or substitute a specific DNA stretch, while NHEJ can generally be employed to disrupt or, conversely, restore the reading frame of a mutated gene [74, 78].

Engineered nucleases developed to date include engineered meganucleases (MNs), zinc finger nucleases (ZFNs), transcription activator-like effector nucleases (TALENs), and clustered regularly interspaced short palindromic repeats (CRISPR), each differing in their way of targeting genomic sequences and mediating double-stranded cleavage [79]. Among them, CRISPR and its associated Cas9 protein has seen widespread adoption due to its ease of design and low cost compared to all previous nuclease families, and therefore it is being widely adopted [74, 80–87]**.**

Three main CRISPR systems, named CRISPR types I, II, and III, have been identified in bacteria and archaea [88] where they function as a form of adaptive immunity to defend themselves against invading phages and plasmids, by using sequence-specific RNA-guided nucleases that cleave foreign genetic elements [88, 89].

CRISPR/Cas9 comes from *Streptococcus pyogenes* and is a type II CRISPR; the most studied among the three existing CRISPR systems. Two main components allow the recognition and cleavage of any genomic region located upstream to a –NGG- triplet named Protospacer Adjacent Motif (PAM): the mature single guide RNA (sgRNA) and the Cas9 endonuclease. Among the 70 nucleotides that define the sgRNA, the 20 nucleotides located at its 5′ are involved in the pairing with the target genomic sequence, while the remaining nucleotides are arranged in a stem loop able to recruit Cas9 [90] The two Cas9 catalytic sites mediate a blunt double strand-break 3 basepairs upstream of the genomic PAM sequence. Specifically, HNH site cleaves the DNA top strand, while the RuvC-like domains operate the cleavage of the bottom strand [91]. Depending on the presence or absence of a DNA template, the cut is re-joined by the intrinsic cell repair mechanisms HDR or NHEJ, previously mentioned, thus introducing a permanent genomic alteration. These repair methods can be selectively exploited depending on the editing to be performed, potentially extending the applicability of this editing tool to any type of genomic mutation. This could translate CRISPR-mediated editing into a suitable therapeutic approach for the treatment of inherited diseases caused by a wide mutational spectrum [92].

### Engineered Nucleases for Dmd Treatment:

As the most common among the muscular dystrophies, the design of approaches targeted to DMD has been a priority in the research field of MDs. Conventional gene-therapy approaches, focusing on the delivery of the corrected gene version to the affected cells, have been attempted in vitro to compensate for the genomic mutation underlying DMD [93, 94]. However, the extremely large size of the dystrophin gene limits its packaging into viral vectors, forcing scientists to adopt miniaturized dystrophin versions lacking more than 50% of the protein sequence, which have only partial functionality [95–97]. Furthermore, the effect of the exogenous DNA could be lost over time if non-integrating viral vectors are chosen for the constructs delivery. On the contrary, direct sequence-specific alteration of the genomic region could allow the endogenous and permanent correction of the genetic defect.

In vitro and in vivo cell-therapy-based approaches tested in DMD patient-derived cells reflected the timeline of the discovery of newer and newer editing tools: MNs, ZFNs, TALENs, and CRISPR have all been used as proof-of-concept approaches to demonstrate the suitability of genome editing to correct DMD myoblasts carrying deletions and out-of-frame mutations in dystrophin mutational hotspots [98–100]. The feasibility of the CRISPR approach has also been demonstrated in induced pluripotent stem cells (iPSC) derived from DMD patient somatic cells, as myotubes obtained from iPSC differentiation expressed functional dystrophin [101]. However, as each of the above-mentioned approaches aim to target specific mutations, they are only suitable for a limited patient population. Charles Gersbach therefore introduced the multiplexed CRISPR editing. This used a lentiviral vector capable of editing multiple sequences at a time, and was suitable to correct up to 62% of mutations causing DMD [102].

The ability, described by Partridge in late 80s [6], of myoblasts derived from a normal donor to restore dystrophin expression in the mdx mouse model of DMD, could be exploited to alleviate the muscle tissue damage in DMD patients: patients’ myoblasts could in fact be isolated, corrected in vitro and used for autologous transplantation, with the advantage that immunosuppression would not be required and re-administration could be possible (Fig. 1b).

However, myoblast transplantation for muscular dystrophies has limitations: the donor cells mechanism for homing and engraftment are inefficient, and studies conducted in mice showed that the regenerative response of transplanted myoblasts is generally low, as the majority of intra-muscularly transplanted myoblasts die [103]. Also, given the extent of muscle mass and the inaccessibility of most of the affected muscles, the heart and diaphragm included, the choice of the optimal delivery method is critical.

A better solution to ensure the maintenance of restored muscle tissue would be targeting the genetics of satellite cells. The transplantation of freshly isolated satellite cells is more efficient compared to cultured myoblasts [16, 104], and donor satellite cells functionally reconstitute the niche [16]. However, this would not be feasible as a treatment, because it is not possible yet to obtain a sufficient number of freshly isolated cells for the treatment of all the affected muscles.

Regardless of the chosen target cell type, in vitro genome engineering might represent a good starting point to evaluate the specificity of these nucleases through in-depth off-target analysis, suitable to develop further adjustment and improvement of this technology.

Alternatively, in vivo editing could be exploited to systemically deliver the chosen genome editing tool to the affected tissue. However, this approach could introduce a further issue related to the immunogenicity that could derive from both the viral delivery vectors, and the nuclease itself. Since they elicit a low immune response, adeno-associated viruses have been recently used by three different groups to mediate in vivo dystrophin correction in dystrophic mice [105••, 106, 107] and have proven to be an efficient method for genome editing tool delivery, even if the rescued dystrophin expression in satellite cells was quite poor [105••].

Finally, it is important to consider that in vitro and in vivo therapies will be applied to patients that already manifest a dystrophic phenotype, but their applicability so far might still be limited to those in which the damage is not too extensive for the beneficial effect of edited cells to counteract the degenerative process.

A turning point that could allow the body-wide restoration of the genetic defect is in utero genome editing that would correct the mutated gene at early developmental stages and potentially abolish muscle degeneration. Ideally, in the case of known familial inheritance of such particular genetic disorders, editing of zygotes would be the best solution, since every cell of the body would carry the restored genetic heritage. Embryos targeted in later stages could, in fact, generate mosaics of corrected and uncorrected cells according to the stage of nuclease delivery and, as the percent of edited cells would be variable, only muscles derived from efficiently targeted cells would express the restored protein. However, it is important to consider that, due to the high degree of spontaneous mutations that can arise, particularly in large genes such as dystrophin, identification of all *at risk* pregnancies or indeed affected embryos generated prior to implantation, would be a challenging task.

### The Other Side of Engineered Nucleases: Generation of Muscular Dystrophy Models

Other than for genome correction, engineered nucleases can be exploited to knock-out or knock-in genes associated with muscular dystrophy and thus to generate new in vivo and in vitro models that mimic in the closest possible way the pathological features are observed in humans. Targeted gene disruption can be easily obtained by means of the NHEJ repair pathway that follows the double-strand breaks introduced by the nucleases, while gene knock-in, mediated by the less common HDR pathway, might be trickier, as the donor DNA has to be integrated in a precise site.

With regard to Duchenne muscular dystrophy, CRISPR/Cas9 has been recently used to generate animal models that complement the repertoire of existing models and better reproduce the human disease course; The commonly used mdx mice, in fact, exhibit a much less severe phenotype than patients [108]. Conversely, CXMD dogs exhibit a more similar phenotype to the human condition, but their size and longer lifespan are limiting in terms of research costs [109]. The KO rat model of Duchenne muscular dystrophy recently developed by Nakamura [110], who disrupted the dystrophin gene by designing CRISPR sgRNAs targeted to two dystrophin exons, has shown a longer disease progression compared to mdx mice and could therefore better intersect research requirements.

CRISPR/Cas9 was also used to knock-out dystrophin in larger mammals that share more genetic and physiological similarity with humans compared to rodents: Hong-Hao Yu obtained 60% of dystrophin targeting by delivering CRISPR/Cas9 components into pig embryos [111], from which derived dystrophic pigs characterized by mobility impairment and cardiac involvement, both features that strictly correlate clinically and functionally to those observed in patients. Yongchang Chen instead developed a Rhesus monkey knock-out model in which the CRISPR/Cas9 targeted 87% of dystrophin alleles [112].

Alternatively, to dramatically reduce the costs of maintenance of these in vivo models, cell lines carrying the desired mutation could be generated. Fibroblasts obtained from a healthy donor could be mutated and differentiated towards the myogenic lineage, as with donor myoblasts, and analyzed in vitro. However, this technique would require a skin or a muscle biopsy, which is invasive. Muscle cell lines could be also obtained from iPSCs, though this may be limited due to lengthy generation time and outcomes that are dependent on differences in culturing conditions. Recent works opened the path to alternative methods to avoid invasive procedures such as biopsy. Ellis Kim and colleagues found out that a subset of epithelial cells isolated from urine are inclined to differentiate into the myogenic lineage and, by using CRISPR/Cas9, succeeded in the disruption of SGCG gene, mutated in a particular muscular dystrophy subtype [113]. The source of this cell type is easily accessible and would ideally allow the generation of libraries with specific gene mutations.

The generation of disease models with genome editing includes all the drawbacks that come with its editing properties: usually both alleles of a gene need to be targeted in order to obtain the desired change, and off-target effects that might occur in other genomic regions could interfere with the phenotypic outcome.

## Editing of the Human Genome: Ethical Consideration

Genome editing technologies hold future therapeutic potential for many inherited genetic disorders including the muscular dystrophies. A promising area of therapeutic development is somatic cell therapy, which is already in various stages of clinical trials for a number of disorders.

Due to its wide applicability and ease of customization, genome engineering has caused unease among many in the scientific community who have expressed fears that alterations beyond somatic cells may have unknown consequences for future generations.

Despite widespread uptake of CRISPR-based gene engineering and programmable nucleases in molecular biology, the technology is still in its infancy. Though progress has been rapid, off-target effects of the endonucleases are still a concern. The human genome could potentially contain sequences equal to or with great similarity to the chosen guide sequence, leading to unwanted off-target effects that could cause the disruption or deregulation of other genes, perturbing the individual genetic heritage. To date, very little evidence exists about the silencing of nucleases inside cells. Moreover, with respect to the differential nuclease efficiency in different cells, each clone may have a peculiar mosaic distribution of off-target effects.

Heritable genetic engineering is of largest concern to most regarding the unknown scope of the changes that could be made; indeed, a moratorium on germline genome editing was suggested after the results shown in two separate studies carried out on non-viable human embryos [114, 115]. The important off-target changes detected in egg, sperm, or embryo DNA, emphasized the fears raised for genome editing before birth, and opened the path to the moral question of “engineered babies”: the possible misuse of genome editing could lead to the eugenic selection of favorable traits in human species. Doubts have also been expressed on the use of human embryos for research, in view of their status of humans.

These issues seem to be overcome if genome editing is meant to investigate the absolute beginning of human life: scientists working in UK obtained the approval for the use of CRISPR to inactivate genes involved in embryo development [116], and similarly, several genes have been modified in four-cells healthy human embryos from Swedish scientists.

## Conclusions

Genome editing technologies are quickly developing to overcome the limitations imposed by classical gene and cell-therapies and could be adopted as a therapeutic approach for degenerative neuromuscular disorders such as muscular dystrophies. Targeting of the stem cell population responsible for the regeneration of muscle tissue, known as satellite cells, seems a possible way to ensure the healing of the muscle fiber from an early stage.

To date, none of the classic genome editing tools provides a suitable solution for the development of effective therapies for muscular dystrophies, both because muscle tissue is too vast to be accessed entirely, and because the nuclease delivery and safety still have to be assessed and optimized.

Genome editing in the zygote would partially limit the issue related to muscle mass extent and satellite cell accessibility, but would not help the muscular dystrophies cases arising due to de novo mutations. The discovery of even more specific editing nucleases combined with extensive studies on the muscle precursors may help the regenerative medicine field in taking a step forward to design an effective cure for these devastating disorders.
